# Association between the TyG index and TG/HDL-C ratio as insulin resistance markers and the risk of colorectal cancer

**DOI:** 10.1186/s12885-022-10100-w

**Published:** 2022-09-22

**Authors:** Tong Liu, Qingsong Zhang, Yiming Wang, Xiangming Ma, Qi Zhang, Mengmeng Song, Liying Cao, Hanping Shi

**Affiliations:** 1 Department of Gastrointestinal Surgery/Clinical Nutrition, Capital Medical University Affiliated Beijing Shijitan Hospital, Beijing, 100038 China; 2 Beijing International Science and Technology Cooperation Base for Cancer Metabolism and Nutrition, Beijing, 100038 China; 3 Key Laboratory of Cancer FSMP for State Market Regulation, Beijing, 100038 China; 4 Department of General Surgery, Kailuan General Hospital, Tangshan, China; 5 Department of Hepatological Surgery, Kailuan General Hospital, Tangshan, 063000 China

**Keywords:** Insulin resistance, Marker, Colorectal cancer, Prospective, Cohort

## Abstract

**Background:**

No previous prospective research has explored the association of the TyG (fasting triglyceride-glucose) index and TG/HDL-C ratio as insulin resistance markers with the risk of colorectal cancer (CRC) incidence in the Northern Chinese population.

**Methods:**

In this prospective cohort study, we included 93,659 cancer-free participants with the measurements of TyG index and TG/HDL-C ratio. Participants were divided by the quartiles of the TyG index or TG/HDL-C ratio. The associations of TyG index, TG/HDL-C ratio, and their components with CRC risk were assessed using Cox proportional hazards regression models.

**Results:**

During a median follow-up of 13.02 years, 593 incident CRC cases were identified. Compared with the lowest quartile of the TyG index (Q1), the risk of CRC was higher in persons in the third (Q3) and highest quartiles (Q4) of the TyG index, with corresponding multivariable-adjusted HRs (95% CI) of 1.36 (1.06, 1.76) and 1.50 (1.19, 1.91), respectively. The elevated risks of CRC incidence were observed in people in the second, third, and highest quartiles of the TG/HDL-C ratio groups, with corresponding multivariable-adjusted HRs (95% CI) of 1.33 (1.05, 1.70), 1.36 (1.07, 1.73) and 1.37 (1.07, 1.75), respectively.

**Conclusions:**

Elevated TyG index and TG/HDL-C ratio were associated with a higher risk of developing CRC among adults in Northern China.

Colorectal cancer (CRC) is one of the most frequent cancers in both men and women and is responsible for over 10% of all cancer cases worldwide [1]. More than 65% of new cases occur in nations with high or very high levels of human development, with Europe and the Americas accounting for half of the estimated new cases [2]. Colorectal cancer incidence is growing in China, and its temporal progression is a crucial indicator of human development changes [3]. Studies have determined that consumption of red meat and alcoholic beverages [4] and lack of physical activity [5] are established causes of CRC, although the underlying causative biological processes have not been clearly explained. In addition, epidemiological studies have indicated that waist circumference (WC) is more significantly associated with CRC risk than body mass index (BMI), highlighting the etiological importance of abdominal or visceral fat disposition rather than total adiposity [6, 7].

As the accumulation of visceral fat is a strong predictor of insulin resistance (IR) and hyperinsulinemia [8], the apparent link between obesity and the risk of CRC has recently been explained by the insulin hypothesis [9]. In line with the insulin hypothesis, epidemiological data tend to suggest a positive association between CRC and IR indicators [10]. For diagnosing IR, the hyperinsulinemic-euglycemic glucose clamp method is the gold standard [11]. .However, this method is time-consuming, expensive, labor-intensive, and technically difficult [12]. It may therefore be valuable and cost-effective for clinicians to use a simple, widely available marker for predicting IR in everyday clinical practice. Previous studies have demonstrated that the fasting triglyceride-glucose (TyG) index and the triglyceride to high-density lipoprotein cholesterol (TG/HDL-C) ratio are closely related to IR, and may serve as markers of IR [13, 14]. The positive association of the TyG index with CRC risk has been reported in previous studies conducted on the Western population [15, 16]. However, whether the IR indicators including TyG index and TG/HDL-C ratio elevate the risk of CRC incidence in the Chinese population is still unclear.

The Kailuan study is an ongoing, prospective cohort study with biennial follow-ups. The measurements of the TyG index and TG/HDL-C ratio components offer us an excellent chance to investigate whether IR (as defined by the TyG index or TG/HDL-C ratio) is associated with the risk of CRC incidence.

## Materials and methods

### Study design and population

The data of this current study came from the Kailuan study (Chinese Clinical Trial Registry number: ChiCTR-TNRC-11001489), which is a prospective cohort study of current and retired Kailuan Group employees in Tangshan City, Hebei Province, northern China [17]. The Kailuan study’s goal was to investigate risk factors for chronic diseases such as cancer. In summary, between July 2006 and October 2007, 101,510 people (81,110 men and 20,400 women, ages 18 to 98 years) were enrolled in the baseline examination, which included a standardized questionnaire survey, physical examinations, and laboratory tests. All participants were subjected to further biennial examinations of the aforementioned measurements.

Participants were excluded for the following reasons: 1) prior cancer diagnosis (*n* = 377); 2) missing data on TyG index and TG/HDL-C ratio measures (*n* = 1342), comprising triglyceride (TG, in mmol/L), fasting blood glucose (FBG, in mmol/L), and high-density lipoprotein cholesterol (HDL-C, in mmol/L); and 3) missing data on other confounders, such as age, sex, body mass index (BMI, in kg/m^2^), total cholesterol levels (TC, in mmol/L), high-sensitivity C-reactive protein levels (hs-CRP, in mg/L), diabetes mellitus, family income of each member, educational background, marital status, smoking status, drinking status, physical activity, sedentary lifestyle, tea consumption, high-fat diets, and family history of cancer (*n* = 6132).

**Fig. 1:**
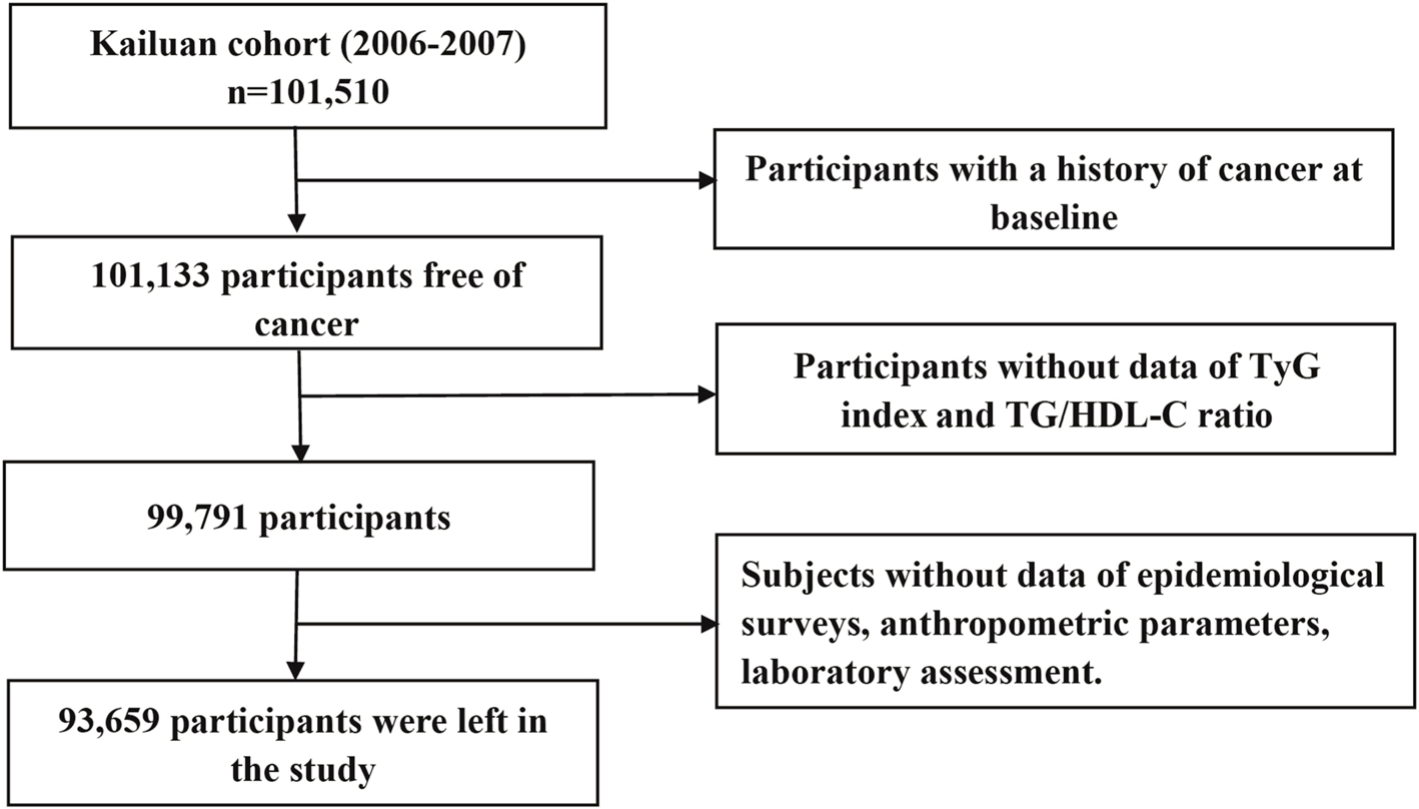
The procedure of participant screening

The final analyses comprised 93,659 individuals, including 18,988 women (20.27%) and 74,671 men (79.73%). Figure 1 depicts the screening details for the participants. The protocol for this study adhered to the Helsinki Declaration criteria and was approved by the ethics committees of Kailuan General Hospital and Beijing Shijitan Hospital. All of the participants signed a written informed consent form.

### Collection and definitions of insulin resistance

After an eight-hour fast, blood samples were collected from the individuals in vacuum tubes containing EDTA, separated, and stored at − 80 °C for subsequent examination. At Kailuan General Hospital’s central laboratory, all laboratory parameters were measured using an autoanalyzer (Hitachi 747; Hitachi, Tokyo, Japan). TG was measured by the colorimetric enzymatic method (Mind Bioengineering Co Ltd., Shanghai, China) with the detectable upper limit being 11.30 mmol/L. The level of HDL-C was measured via the direct test method (Mind Bioengineering Co. Ltd., Shanghai, China) and the detectable upper limit was 3.88 mmol/L. The hexokinase/glucose-6-phosphate dehydrogenase method was used to measure FBG concentrations. The TG/HDL-C ratio and TyG index were estimated using the following calculations: ln [TG (mg/dL) × FBG (mg/dL) /2] [18]. The quartiles of the TG/HDL-C ratio or the TyG index were used to split the participants into 4 groups.

### Ascertaining the outcome

Trained medical staff identified the incident CRC using the following methods: 1) tracking participants when they had clinical examinations every 2 years until December 31, 2019; 2) reviewing medical records from Tangshan medical insurance system and Kailuan Social Security Information System annually, and 3) examining death certificates from the Provincial Vital Statistics Offices (PVSO) once a year to gather additional information. Clinical specialists reviewed medical records and pathology reports to confirm the diagnosis of incident CRC and classified CRC patients as C18–21 using the International Classification of Diseases, Tenth Revision (ICD-10).

### Potential confounders

A standard questionnaire was used to collect information on each participant’s age, sex, socioeconomic situation, educational background, living habits, and personal and family members’ medical histories by trained medical staff. WC was measured with a tape measure midway between the lowest rib and the pelvis while the participant exhaled. Abdominal obesity was defined as WC ≥ 95 cm in men and ≥ 90 cm in women. Diabetes mellitus was diagnosed as follows: an FPG level ≥ 7.0 mmol/L, use of oral hypoglycemic medications or insulin, or a validated physician diagnosis. Hypertension was defined as an SBP ≥ 140 mmHg, a DBP ≥ 90 mmHg, or a self-reported history of hypertension.

### Statistical analysis

To express and compare normally distributed variables, the mean ± standard deviation and t-test were utilized. The skewed distribution (hs-CRP and TG) variables were described and compared using the median (interquartile range) and a nonparametric test (Kruskal-Wallis test). Absolute values with percentages and the chi-square test were used to express and compare categorical variables. The discriminatory power of various IR indicators as well as their components for the development of CRC was tested with the area under the receiver-operating characteristic (ROC) curve. The calculation of person-years began on the date of the baseline examination and continued through the date of CRC diagnosis, death, or the 31st of December 2019. The dose-response association of the TyG index and TG/HDL-C ratio with CRC risk was calculated by restricted cubic spline regression (RCS). The proportional hazard assumption was checked using Schoenfeld residual test. The hazard ratios (HRs) and their 95% confidence intervals (CIs) for the TyG index, TG/HDL-C ratio, or their components associated with the risk of incident CRC were calculated by the Cox regression. Subgroup analyses were conducted by stratifying participants by sex (male vs. female), age (≤45 years, 45–65 years, > 65 years), abdominal obesity, and diabetes mellitus.

To rule out the potential of reverse causality, we removed participants who developed cancer during the first year of follow-up from the sensitivity analysis. We also removed patients who took statins, oral hypoglycemic medications, or insulin to rule out any possible influence of medication on the TyG index or TG/HDL-C ratio levels. We further excluded participants with extreme TyG index or TG/HDL-C ratio values (< 1% and > 99%) to check the robustness of the results.

A *p*-value (two-sided) < 0.05 was considered statistically significant. Statistical analyses were carried out with the help of a commercially available software tool (SAS software, version 9.4).

## Results

Table 1 shows the individuals’ baseline characteristics stratified by sex. The average age of the study population was 51.44 ± 12.45 years. There were significant differences in age, levels of FBG, HDL-C, TG, TC, hs-CRP, BMI, WC, TyG index, and TG/HDL-C ratio. Furthermore, the percentages of reported income, marital status, educational levels, physical exercise, sedentary lifestyle, smoking status, drinking status, high-fat diets, salt intake, hypertension, and diabetes mellitus varied significantly across the male and female groups. However, there was no difference in the prevalence of a family history of cancer between the two groups.

**Table 1:** Baseline characteristics of the participants stratified by sex

Variables	Overall	Women	Men	***P***-value
**n (%)**	93,659	18,988	74,671	
**Age (year)**	51.44 ± 12.45	48.67 ± 11.46	52.24 ± 12.60	**< 0.001**
**FBG (mmol/L)**	5.48 ± 1.68	5.32 ± 1.63	5.52 ± 1.69	**< 0.001**
**HDL-C (mmol/L)**	1.55 ± 0.40	1.59 ± 0.39	1.54 ± 0.39	**< 0.001**
**TG (mmol/L)**	1.27(0.90,1.93)	1.18(0.82,1.75)	1.30(0.92,1.98)	**< 0.001**
**TC (mmol/L)**	4.95 ± 1.15	4.98 ± 0.06	4.94 ± 0.10	**< 0.001**
**Hs-CRP (mg/L)**	0.80(0.30,2.06)	0.80(0.30,2.28)	0.80(0.30,2.00)	**0.002**
**BMI (Kg/m**^**2**^**)**	25.05 ± 3.50	24.66 ± 3.81	25.15 ± 3.41	**< 0.001**
**WC (cm)**	86.90 ± 9.99	82.89 ± 10.70	87.92 ± 9.53	**< 0.001**
**TyG index**	8.66 ± 0.69	8.53 ± 0.68	8.69 ± 0.69	**< 0.001**
**TG/HDL-C ratio**	0.85(0.57,1.36)	0.76(0.51,1.17)	0.88(0.59,1.41)	**< 0.001**
**Reported income (¥)**				**< 0.001**
**< 600**	27,001(28.83)	3593(18.92)	23,408(31.35)	
**600–800**	53,246(56.85)	12,411(65.36)	40,835(54.69)	
**800–1000**	7170(7.66)	1437(7.57)	5733(7.68)	
**> 1000**	6242(6.66)	1547(8.15)	4695(6.29)	
**Marital status (%)**				**< 0.001**
**Never**	1586(1.69)	358(1.89)	1228(1.64)	
**Married**	88,369(94.35)	17,645(92.93)	70,724(94.71)	
**Divorced**	803(0.86)	264(1.39)	539(0.72)	
**Widowed**	1920(2.05)	539(2.84)	1381(1.85)	
**Remarried.**	981(1.05)	182(0.96)	799(1.07)	
**Educational background (%)**				**< 0.001**
**Never**	1143(1.22)	81(0.43)	1062(1.42)	
**Primary school**	8981(9.59)	874(4.60)	8107(10.86)	
**Middle school**	64,837(69.23)	12,546(66.07)	52,291(70.03)	
**High school**	12,230(13.06)	3404(17.93)	8826(11.82)	
**College graduate or above**	6468(6.91)	2083(10.97)	4385(5.87)	
**Physical exercise (%)**				**< 0.001**
**Never**	8138(8.69)	893(4.70)	7245(9.70)	
**Occasionally**	70,873(75.67)	15,550(81.89)	55,323(74.09)	
**Regularly**	14,648(15.64)	2545(13.40)	12,103(16.21)	
**Smoking status (%)**				**< 0.001**
**Never**	56,067(59.86)	18,549(97.69)	37,518(50.24)	
**Past**	5324(5.68)	82(0.43)	5242(7.02)	
**Moderate**	3320(3.54)	89(0.47)	3231(4.33)	
**Severe**	28,948(30.91)	268(1.41)	28,680(38.41)	
**Drinking status (%)**				**< 0.001**
**Never**	55,263(59.00)	17,670(93.06)	37,593(50.34)	
**Past**	3624(3.87)	80(0.42)	3544(4.75)	
**Moderate**	18,012(19.23)	1144(6.02)	16,868(22.59)	
**Severe**	16,760(17.89)	94(0.50)	16,666(22.32)	
**Sedentary lifestyle (%)**				**< 0.001**
**< 4 hours/day**	70,028(74.77)	13,997(73.71)	56,031(75.04)	
**4–8 hours/day**	20,593(21.99)	4281(22.55)	16,312(21.85)	
**> 8 hours/day**	3038(3.24)	710(3.74)	2328(3.12)	
**High-fat diets (%)**				**< 0.001**
**Seldom**	7936(8.47)	1817(9.57)	6119(8.19)	
**Occasionally**	77,097(82.32)	16,233(85.49)	60,864(81.51)	
**Regularly**	8626(9.21)	938(4.94)	7688(10.30)	
**Salt intake (%)**				**< 0.001**
**Low (< 6 g/day)**	8637(9.23)	1764(9.30)	6873(9.21)	
**Intermediate (6–10 g/day)**	74,886(80.00)	15,925(83.92)	58,961(79.00)	
**High (> 10 g/day)**	10,088(10.78)	1288(6.79)	8800(11.79)	
**Family history of cancer (%)**	3428(3.66)	867(4.57)	2561(3.43)	**0.828**
**Diabetes mellitus (%)**	8501(9.08)	1480(7.79)	7021(9.40)	**< 0.001**
**Hypertension (%)**	40,861(43.63)	6075(31.99)	34,786(46.59)	**< 0.001**

*Hs-CRP* high-sensitivity C-reactive protein, *FBG* fasting blood glucose *HDL-C* high-density lipoprotein cholesterol, *TG* triglyceride, *WC* waist circumference, *TC* total cholesterol, *AVI* visceral adiposity index, *TyG* fasting triglyceride-glucose

**Fig. 2:**
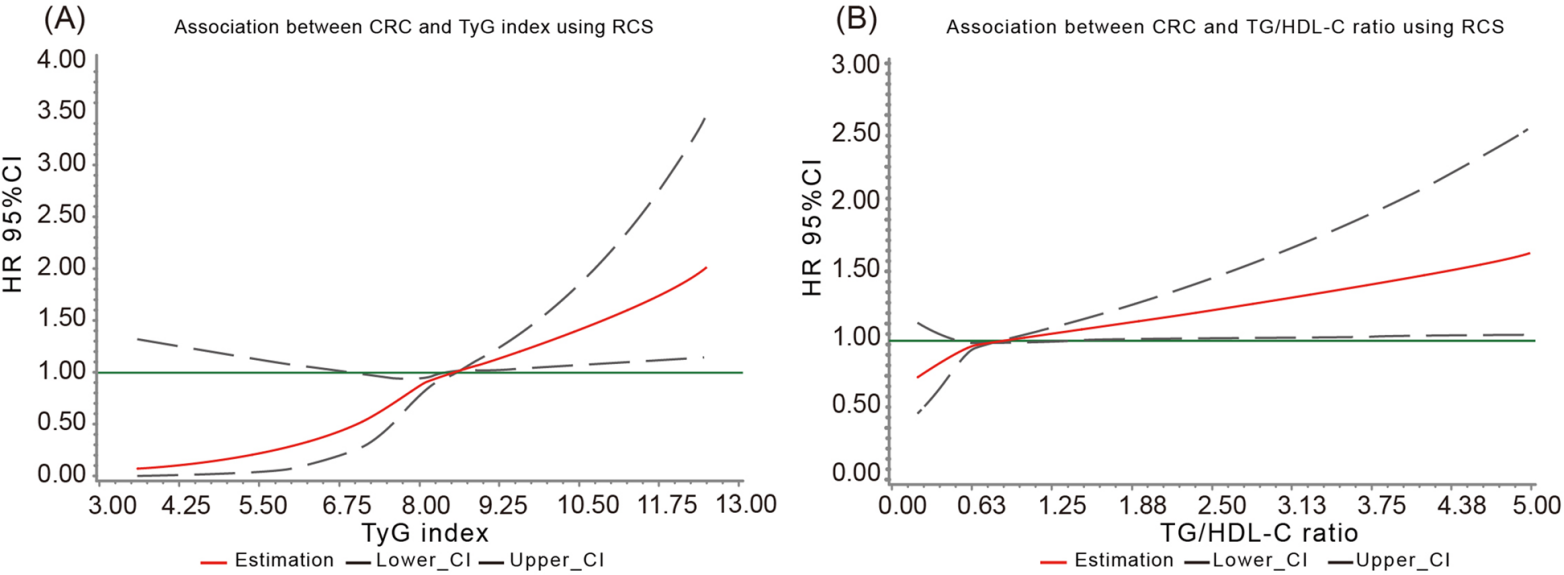
Association of the TyG index and TG/HDL-C ratio with CRC risk using RCS with 3 knots. Cubic spline graph of the adjusted HR (represented by solid line) and 95% CI (represented by the dotted line)

The median (IQR) duration of follow-up was 13.02 (12.69, 13.20) years, 593 incident CRC cases were identified at the end of follow-up. The results from RCS (Fig. 2) showed a positive dose-response but nonlinear association of the TyG index (*P*-overall< 0.001, *P*-nonlinear = 0.033) and TG/HDL-C ratio (*P*-overall = 0.008, *P*-nonlinear = 0.011) with CRC risk. The proportional hazard assumption was verified by the Schoenfeld residual global test (*P* = 0.322). Table 2 shows the crude and adjusted HRs (95% CI) for the association of quartiles of the TyG index and TG/HDL-C ratio with the risk of CRC. Compared with the lowest quartile of the TyG index (Q1), the risk of CRC was higher in persons in the third (Q3) and highest quartiles (Q4) of the TyG index, with corresponding HRs (95% CI) of 1.43 (1.12–1.83) and 1.53 (1.20, 1.93), respectively, in the adjusted models. Similarly, statistically significant increased risks of CRC incidence were observed in people in the second, third, and highest quartiles of the TG/HDL-C ratio groups, with corresponding HRs (95% CI) of 1.33 (1.05, 1.70), 1.37 (1.08, 1.75) and 1.40 (1.09, 1.78), respectively. Similar results were also obtained in the diabetes-adjusted models. We also examined the association between IR indicator components, including TG, FBG, HDL-C, and CRC risk (Table 3). Positive associations of TG and FBG with CRC risk were observed in the adjusted models. And a reversed association of HDL-C with CRC was found in the multivariate analyses for the second versus the lowest quartile of HDL-C, however, the inverse effect did not exist with the increase of HDL-C. This finding was partly in line with a previous study using data from the Kailuan Study where the protective effect of HDL-C against the occurrence of stroke was observed when HDL-C was less than 1.42 mmol/L. [19]

**Table 2:** Hazard ratios (HRs) for the association of TyG index or TG/HDL-C ratio with CRC risk

Group	Cases/person-years	Crude		Adjusted ^**a**^		Diabetes-adjusted ^**b**^	
		HR (95%CI)	***p***-value	HR (95%CI)	***p***-value	HR (95%CI)	***p***-value
**TyG index (per 1 unit)**	593/1145910	**1.27(1.13,1.41)**	**< 0.001**	**1.21(1.08,1.36)**	**0.001**	**1.19(1.05,1.34)**	**0.008**
**Quartiles of TyG index**							
**Q1 (≤8.19)**	111/290016	Ref.		Ref.		Ref.	
**Q2 (8.19 ~ 8.58)**	131/287327	1.19(0.93,1.53)	0.174	1.13(0.88,1.46)	0.339	1.13(0.88,1.45)	0.351
**Q3 (8.58 ~ 9.06)**	170/282736	**1.57(1.24,1.99)**	**< 0.001**	**1.43(1.12,1.83)**	**0.004**	**1.36(1.06,1.76)**	**0.017**
**Q4 (≥9.06)**	181/285831	**1.65(1.31,2.09)**	**< 0.001**	**1.53(1.20,1.93)**	**0.001**	**1.50(1.19,1.91)**	**0.001**
***p*for trend**			**< 0.001**		**0.001**		**0.004**
**TG/HDL-C ratio (per 1 unit)**	593/1145910	**1.13(1.03,1.23)**	**0.006**	**1.12(1.02,1.22)**	**0.016**	**1.11(1.01,1.21)**	**0.027**
**Quartiles of TG/HDL-C ratio**							
**Q1 (≤0.57)**	113/283296	Ref.		Ref.		Ref.	
**Q2 (0.57 ~ 0.85)**	153/289407	**1.33(1.04,1.69)**	**0.023**	**1.33(1.05,1.70)**	**0.021**	**1.33(1.04,1.70)**	**0.023**
**Q3 (0.85 ~ 1.36)**	162/286268	**1.42(1.11,1.80)**	**0.005**	**1.37(1.08,1.75)**	**0.010**	**1.36(1.07,1.73)**	**0.013**
**Q4 (≥1.36)**	165/286939	**1.44(1.13,1.82)**	**0.003**	**1.40(1.09,1.78)**	**0.008**	**1.37(1.07,1.75)**	**0.013**
***p*for trend**			**0.014**		**0.032**		**0.045**

Results presented with bold valued were statistically significant with all *p* values < 0.05

^a^ Adjusted for age (every 10 years), sex, family income, educational background, marital status, WC, TC, smoking status, drinking status, physical activity, sedentary lifestyle, tea consumption, salt intake, high-fat diet, hypertension, and family history of cancer

^b^ Further adjusted for diabetes

**Table 3:** Hazard ratios (HRs) for the association of TG, FBG or HDL-C with CRC risk

Group	Cases/person-years	Crude models		Adjusted models	
		HR (95%CI)	***p***-value	HR (95%CI)	***p***-value
**TG (continuous, per SD)**	593/1145910	1.05(1.00,1.11)	0.051	1.05(0.99,1.10)	0.089
**Quartiles of TG**					
**Q1 (≤0.90)**	109/294585	Ref.		Ref.	
**Q2 (0.90 ~ 1.27)**	142/281968	**1.36(1.06,1.75)**	**0.016**	1.30(1.01,1.67)	0.041
**Q3 (1.27 ~ 1.93)**	179/284244	**1.70(1.34,2.15)**	**< 0.001**	**1.58(1.24,2.01)**	**< 0.001**
**Q4 (≥1.93)**	163/285114	**1.54(1.21,1.96)**	**0.017**	**1.45(1.13,1.85)**	**0.003**
***p*for trend**			**< 0.001**		**0.002**
**FBG (continuous, per SD)**	593/1145910	**1.06(1.02,1.10)**	**0.006**	**1.05(1.01,1.09)**	**0.009**
**Definition of diabetes**^**a**^					
**None (< 6.1 mmol/L)**	144/289661	Ref.		Ref.	
**IFG or IGT (6.1 ~ 7.0)**	128/290728	1.23(0.93,1.63)	0.141	1.09(0.83,1.44)	0.538
**Diabetes (≥ 7.0 mmol/l)**	188/280042	**1.59(1.24,2.05)**	**< 0.001**	**1.33(1.03,1.72)**	**0.029**
***p*for trend**			**< 0.001**		0.086
**HDL-C (continuous, per SD)**	593/1145910	1.21(0.99,1.47)	0.059	1.09(0.89,1.32)	0.402
**Quartiles of HDL-C**					
**Q1 (≤1.28)**	162/290143	Ref.		Ref.	
**Q2 (1.28 ~ 1.51)**	126/293293	**0.77(0.61,0.97)**	0.027	**0.77(0.61,0.97)**	**0.028**
**Q3 (1.51 ~ 1.77)**	131/285994	0.82(0.65,1.03)	0.093	0.82(0.65,1.03)	0.088
**Q4 (≥1.77)**	174/276480	1.12(0.91,1.39)	0.288	1.02(0.82,1.27)	0.856
***p*for trend**			**0.003**		**0.031**

Adjusted models include age (every 10 years), sex, family income, educational background, marital status, WC, TC, smoking status, drinking status, physical activity, sedentary lifestyle, tea consumption, salt intake, high-fat diet, hypertension, and family history of cancer

Results presented with bold valued were statistically significant with all *p* values < 0.05

^a ^*IFG *impaired fasting glucose, *IGT *impaired glucose tolerance

**Fig. 3:**
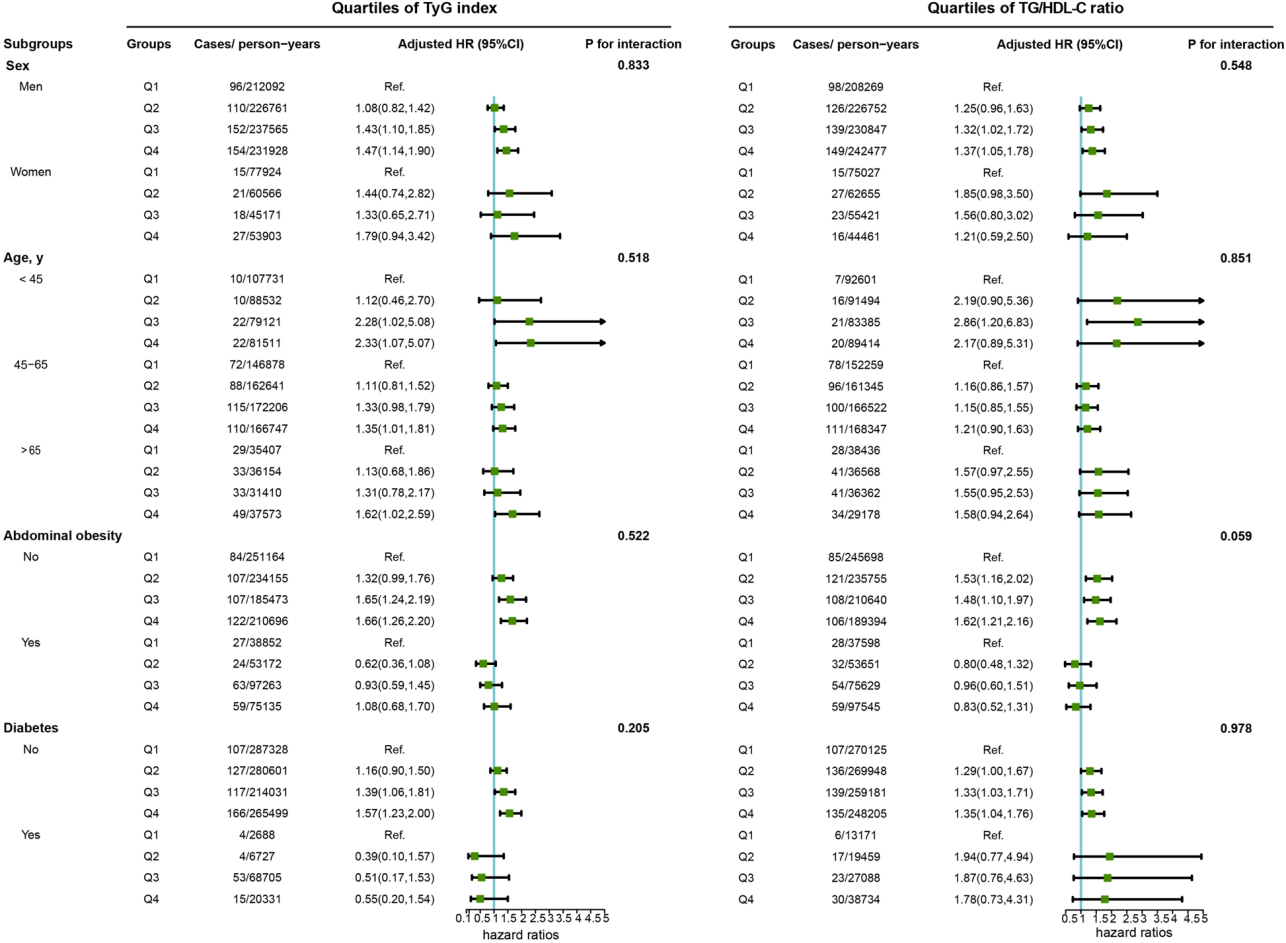
Subgroup analysis of the association of the TyG index and TG/HDL-C ratio with CRC risk. Note: Models were adjusted for age (every 10 years), sex, family income, educational background, marital status, BMI, TC, smoking status, drinking status, physical activity, sedentary lifestyle, tea consumption, salt intake, high-fat diet, hypertension, diabetes, and family history of cancer

Figure 3 illustrates the subgroup analyses by sex, age, abdominal obesity, and diabetes. Elevated levels of the TyG index were associated with an increased risk of CRC incidence among participants who were male, young (< 45 y), middle-aged (45–65 y), elderly (≥65 y), and without abdominal obesity or diabetes in the multivariate analyses. In addition, positive associations between the TG/HDL-C ratio and CRC risk were observed among men, young (< 45 y), without abdominal obesity or diabetes groups.

**Fig. 4:**
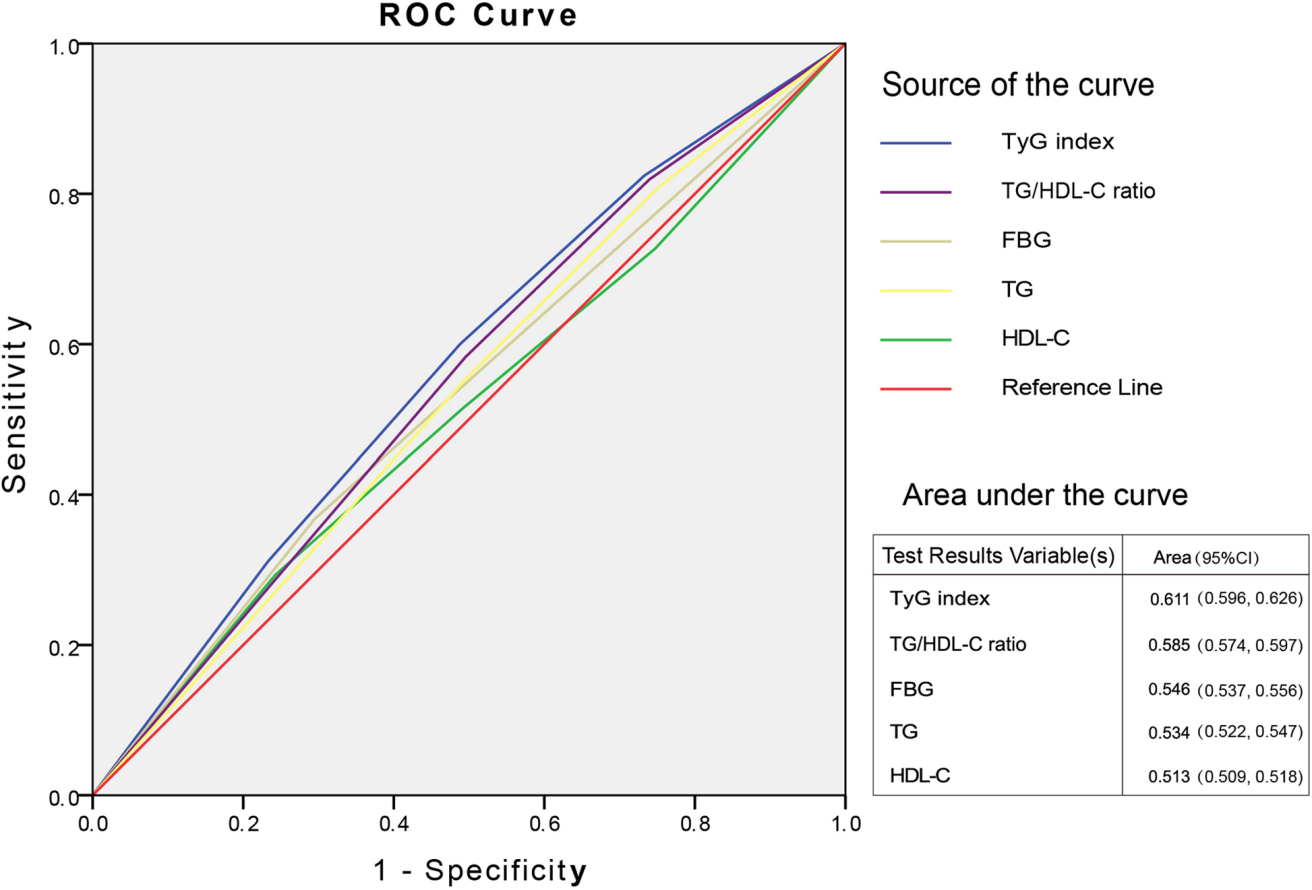
The discriminatory power of various IR indicators as well as their components for the development of CRC

Figure 4 illustrates the area under the ROC curve. The TyG index had the highest level of the area under the ROC curve (AUC = 0.611, 95%CI: 0.596, 0.626), followed by TG/HDL-C ratio (AUC = 0.585, 95%CI: 0.574, 0.597), FBG (AUC = 0.546, 95%CI: 0.537, 0.556), TG (AUC = 0.534, 95%CI: 0.522, 0.547), and HDL-C (AUC = 0.513, 95%CI: 0.509, 0.518).

In the sensitivity analyses, after excluding 26 participants who developed CRC within the 1st year of follow-up, 911 individuals who took statins at baseline, 2365 participants who received oral hypoglycemic agents or insulin, 1881 or 1893 participants with extreme values of TyG index or TG/HDL-C ratio, significant associations of the TyG index and TG/HDL-C ratio with the risk of CRC incidence were still observed (Table 4).

**Table 4:** Sensitivity analyses of the association of TyG index or TG/HDL-C ratio with CRC risk

	TyG index		TG/HDL-C ratio	
	HR (95%CI)	***p***-value	HR (95%CI)	***p***-value
**Exclude CRC occurred within 1 year**				
**Q1**	Ref.		Ref.	
**Q2**	1.13(0.87,1.46)	0.354	**1.31(1.02,1.68)**	**0.035**
**Q3**	**1.40(1.10,1.80)**	**0.007**	**1.39(1.08,1.78)**	**0.011**
**Q4**	**1.47(1.16,1.88)**	**0.001**	**1.35(1.04,1.74)**	**0.023**
**Exclude participants who took statins**				
**Q1**	Ref.		Ref.	
**Q2**	1.15(0.89,1.49)	0.278	**1.35(1.05,1.72)**	**0.018**
**Q3**	**1.46(1.14,1.87)**	**0.002**	**1.38(1.08,1.77)**	**0.011**
**Q4**	**1.56(1.23,1.98)**	**< 0.001**	**1.40(1.09,1.80)**	**0.009**
**Exclude participants who received oral hypoglycemic agents or insulin**				
**Q1**	Ref.		Ref.	
**Q2**	1.13(0.87,1.46)	0.363	**1.31(1.02,1.68)**	**0.034**
**Q3**	**1.44(1.12,1.84)**	**0.004**	**1.37(1.07,1.76)**	**0.013**
**Q4**	**1.53(1.20,1.95)**	**0.001**	**1.36(1.05,1.75)**	**0.020**
**Exclude participants with extreme values of TyG index or TG/HDL-C ratio**				
**Q1**	Ref.		Ref.	
**Q2**	1.12(0.86,1.44)	0.371	**1.33(1.04,1.71)**	**0.020**
**Q3**	**1.42(1.10,1.82)**	**0.005**	**1.37(1.08,1.77)**	**0.012**
**Q4**	**1.50(1.19,1.94)**	**0.001**	**1.34(1.04,1.73)**	**0.030**

Adjusted models were adjusted for age (every 10 years), sex, family income, educational background, marital status, WC, TC, smoking status, drinking status, physical activity, sedentary lifestyle, tea consumption, salt intake, high-fat diet, hypertension, diabetes mellitus, and family history of cancer

Results presented with bold valued were statistically significant with all *p* values < 0.05

## Discussion

To our knowledge, this study is the first prospective cohort study to investigate the association of IR indicators, including the TyG index and TG/HDL-C ratio, with the risk of CRC incidence in the Northern Chinese population. We found participants with elevated levels of TyG index and TG/HDL-C ratio exhibited a higher risk of CRC incidence. Subgroup analyses and sensitivity analyses further validated the robustness of the primary results. In addition, the TyG index outperformed the TG/HDL-C ratio as well as their components for predicting the future development of CRC.

We discovered that both the TyG index and the TG/HDL-C ratio were associated with an increased risk of CRC incidence, which was partly consistent with a previous cohort study. Takuro Okamura et al. reported that the TyG index is an effective and accessible tool for predicting incident CRC by analyzing data from 27,944 individuals (16,454 men and 11,490 women) [15]. By analyzing 510,471 individuals from six European cohorts, Josef Fritz et al. found TyG index was associated with an increased risk of gastrointestinal cancers [16]. However, no research has been conducted on the influence of the TG/HDL-C ratio on the incidence of CRC. Several epidemiological studies have proven a strong link between IR and CRC risk. Hamid Farahani et al. recently found that CRC patients had a higher HOMA-IR than controls in a case-control study, including 88 controls and 82 cases with CRC [20]. In a nested case-control study, Paul J. Limburg et al. found that HOMA-IR was significantly associated with incident CRC based on a comparison of the highest versus the lowest quartiles of HOMA-IR [21]. A meta-analysis including 8 studies involving 2956 patients and 347,326 total participants to assess the association between HOMA-IR and the risk of CRC found that higher levels of HOMA-IR were significantly associated with an increased risk of CRC (pooled OR = 1.47, 95% CI 1.24 to 1.74) and remained significant in all subgroups, including for sex, study design and geographic region [22].

We found that the components of the IR index, including TG and FBG, were positively associated with an elevated risk of CRC incidence, which was consistent with earlier research. By analyzing data from 156,153 subjects from a health investigation (1988–2003), H Ulmer et al. found that TG concentrations were associated with an increased risk of rectal cancer [23]. A prospective cohort study conducted by Tanja Stocks et al. found a significant association between TG and CRC risk in men in the Metabolic Syndrome and Cancer Project (Me-Can) [24]. However, several studies failed to find such a relationship [25, 26]. The results regarding the association of FBG with CRC incidence were also consistent with prior research. In a Korean investigation, CRC incidence was found to be positively associated with FBG levels in men. In the site-specific analysis, elevated FBG was especially associated with rectal cancer incidence [27]. By analyzing data from the Framingham Heart Study Offspring Cohort (1971–2008), Niyati Parekh et al. demonstrated that elevated FBG levels were associated with a 2-fold increased risk of colon cancer [28].

Several underlying mechanisms may help elucidate the carcinogenic effect of the TyG index and TG/HDL-C ratio on the development of CRC. First, previous fundamental research found that IR was linked to hyperinsulinemia [29], which activates the PI3K/Akt/mTOR/S6K signaling pathway in cancer. According to previous studies, insulin promotes colon cancer progression by increasing the expression of acyl-coenzyme A: cholesterol acyltransferase-1 [30], and vascular cell adhesion molecule-1 in intestinal tumor endothelial cells, resulting in an inflammatory response that promotes malignancy [31]. Second, IR is linked to increased levels of IGF-1 [32]. IGF-1 stimulates the production of vascular endothelial growth factor, which promotes tumor development [33]. Additionally, IGF-1 stimulates cell proliferation, survival, and angiogenesis by activating the IGF-1 receptor [34]. Third, intranuclear NF-κB, which regulates cell proliferation, neoplasia, and metastasis, is reported to be increased by hyperglycemia in humans. Fourth, a previous study demonstrated the association between PPARγ and IR in adipocytes [35]. The close association between PPARγ and CRC has been reported by several studies [36, 37].

Among the main strength of this study is that it provides new evidence for the potential association between IR and cancer incidence. The present study also assesses a wide range of confounding factors, such as lifestyle habits and cancer-associated diseases. Finally, the large sample size, long follow-up, and the prospective design should also be noticed when interpreting the results.

Several limitations should also be recognized in the current study. First, instead of the hyperinsulinemic-euglycemic glucose clamp method, IR was measured using surrogate biomarkers such as the TyG index and the TG/HDL-C ratio. Although the validity of these biomarkers has been reviewed in earlier research, there may be a misclassification of the potential influence of IR on the incidence of CRC. Second, the Kailuan study lacks detailed information on cereal, vegetable, and high-fiber diet intake, which prevents us from examining confounding variables precisely. Socioeconomic variables and lifestyle behaviors were collected via self-reported questionnaires, which may contribute to recall bias. Third, due to the industrial character of the Kailuan community, there is an imbalance in sex distribution. Nonetheless, because we performed a separate statistical study on both sexes, the effect of sex distribution imbalance on the results would be minor. Fourth, due to a lack of precise information, colon cancer, and rectal cancer could not be analyzed separately. IR may have different carcinogenic effects on the occurrence of colon and rectal cancers. Fifth, we also explored the carcinogenic effects of IR indicators on the development of other cancer types, however, no positive associations were found in the current study (data not shown). Finally, we only observed a positive association of IR metrics (TyG index, TG/HDL-C ratio) with CRC risk, but no causal relationship, which deserves better exploration in future studies.

## Conclusions

In summary, we found participants with elevated levels of TyG index and TG/HDL-C ratio exhibited a higher risk of CRC incidence. Because associated risk factors may be pathogenically related to the process of the illness, findings from this study may help explain the pathogenesis of CRC and may be used as a clinical screening tool to identify individuals at high risk of developing CRC in the Northern Chinese population.

## Data Availability

The datasets generated and/or analyzed during the current study are not publicly available due to Kailuan policy but are available from the corresponding author on reasonable request.

## Abbreviations

BMI: Body mass index
CIs: Confidence intervals
CRC: Colorectal cancer
FBG: Fasting blood glucose
TyG: Fasting triglyceride-glucose
HDL-C: High-density lipoprotein cholesterol
HRs: Hazard ratios
hs-CRP: High-sensitivity C-reactive protein
TC: Total cholesterol
TG: Triglyceride
WC: Waist circumference
